# Modeling the renoprotective mechanisms of SGLT2 inhibition in hypertensive chronic kidney disease

**DOI:** 10.14814/phy2.15836

**Published:** 2023-11-13

**Authors:** John S. Clemmer, Timothy E. Yen, Yoshitsugu Obi

**Affiliations:** ^1^ Department of Physiology and Biophysics University of Mississippi Medical Center Jackson Mississippi USA; ^2^ Department of Medicine, Division of Nephrology University of Mississippi Medical Center Jackson Mississippi USA

**Keywords:** chronic kidney disease, physiological modeling, SGLT2 inhibitor

## Abstract

Sodium‐glucose cotransporter (SGLT)‐2 inhibitors have recently been approved for chronic kidney disease (CKD) based on their ability to lower proteinuria and slow CKD progression independent of diabetes status. In diabetic renal disease, modulation of tubuloglomerular feedback (TGF) leading to lower intraglomerular pressure has been postulated as one of the mechanisms of renal protection with SGLT2 inhibition; however, this mechanism has not been sufficiently explored in non‐diabetic CKD. We hypothesized that SGLT2 inhibition exerts renoprotection in CKD through increasing TGF despite normoglycemia. To test this hypothesis, we used an integrative mathematical model of human physiology, HumMod. Stage 3 CKD conditions were simulated by reducing nephron mass which was associated with hypertension, low glomerular filtration rate (GFR) (55 mL/min), hyperfiltration of remnant nephrons, elevated albuminuria (500 mg/day), and minimal levels of urinary glucose (0.02 mmol/L). SGLT2 inhibition was associated with acute reductions in GFR associated with afferent arteriolar vasoconstriction due to TGF. After 12 months, glomerular pressure, nephron damage, and chronic GFR decline were reduced with SGLT2 inhibition with additional SGLT1 inhibitory effects further enhancing these effects. This model supports the use of SGLT2 inhibitors to reduce hyperfiltration in CKD and mitigate renal disease progression, even in the absence of diabetes.

## 1 INTRODUCTION

Chronic kidney disease (CKD) affects 10% of the world population and is associated with hypertension (HTN) and increased cardiovascular morbidity and mortality (Jha et al., 2013). HTN in CKD and other hyperfiltering states such as type 2 diabetes (T2D) accelerates the progression of CKD because of the pressure transmitted to the glomeruli (Drawz & Rahman, 2015). A compensatory intraglomerular hyperfiltration of the remaining nephrons can cause proteinuria through capillary and podocyte damage, leading to the further decline in glomerular filtration rate (GFR) (Chagnac et al., 2019). Until the recent FDA approval of sodium‐glucose cotransporter 2 (SGLT2) inhibitors, renin–angiotensin system (RAS) inhibitors were the only pharmacological drug class that have been consistently proven to slow CKD progression and lower the risk of end stage renal disease (ESRD) (Hou et al., 2006; Ruggenenti et al., 1999), and no treatment has been shown to be effective at stopping or reversing CKD progression.

SGLT2 inhibitors block the uptake of sodium and glucose in the proximal tubule and induce natriuresis and glucosuria (Nespoux & Vallon, 2018). SGLT2 inhibitor treatment for T2D has resulted in multifaceted favorable effects on blood glucose, blood pressure (BP), cardiovascular disease, and CKD progression (Hsia et al., 2017). However, the strong renoprotective effects of SGLT2 inhibitors cannot be explained by the improvements in glucose and BP control alone and have been confirmed independent of diabetic status (Heerspink et al., 2020; Jafar, 2021). Current hypotheses for the renoprotective action of SGLT2 inhibitors relate to inflammation (Packer, 2020), hypoxia (Packer, 2021), metabolism (Thomas & Cherney, 2018), and tubuloglomerular feedback (TGF) (Thomson & Vallon, 2021). However, most of these mechanisms have only been tested in diabetic animal models, and the exact mechanisms and direct renal benefits in non‐diabetic CKD remain unclear.

Recent experimental findings from diabetic animal studies suggest that increased glucose delivery to the macula densa may cause glucose uptake through SGLT1, induce nitric oxide production, and alter afferent arteriolar tone leading to hyperfiltration (Song et al., 2019; Thomson & Vallon, 2019; Zhang et al., 2019). However, the role of SGLT1 in TGF or renal hemodynamics in humans has not been explored, and the effect of dual SGLT2/1 inhibition in non‐diabetic CKD is unknown. In this study, we hypothesize that our model can recapitulate the clinical trials (DAPA‐CKD and EMPA‐Kidney) that demonstrate renoprotection and attenuation of GFR decline during chronic non‐diabetic CKD treatment with SGLT2 inhibition (Heerspink et al., 2020; The et al., 2023). We also hypothesized that the hyperglycemia at the macula densa that occurs during SGLT2 inhibition may actually attenuate the renoprotective effects of these drugs if they lack SGLT1 inhibitory effects.

Because examining chronic systemic and glomerular hemodynamics in response to fluctuating plasma glucose and concentrations of SGLT2 inhibitor cannot be achieved experimentally, we used the established mathematical model of human physiology, HumMod (Clemmer et al., 2017; Hester et al., 2011; Moore & Clemmer, 2021). HumMod serves as a framework for understanding complex responses to pathophysiological conditions and can examine complex interactions among physiological variables that may not be intuitive. This model is based on physiological principles and well‐established physiological relationships described below. For the current study, there have been only minor additions to the model in order to simulate SGLT2 inhibition.

## 2 METHODS

### 2.1 Model description

HumMod is an established, integrative mathematical model of human physiology that has evolved considerably from the original model created and developed by Arthur Guyton and associates more over the past 50 years (Guyton, 1980, 1990; Guyton et al., 1972). HumMod accurately reproduces the acute and chronic responses to many physiological perturbations and the findings in many pathophysiological states (Clemmer et al., 2017, 2021; Hester et al., 2011). The current version of HumMod used in this study has minor differences from the model used to generate the simulations presented in a recent publication focusing on calcium channel blockers in CKD (Moore & Clemmer, 2021). Complete detail of the entire model is beyond the scope of this study. However, the model, model code, general instructions on how to use the model, and instructions on how to run the specific simulations presented in the current study are available for academic download as a single ZIP file at http://hummod.org/hummod‐sglt2.zip. This model can only be run on computers with or emulating a Windows operating system. Additional supplementary materials describing other simulations can be downloaded at https://doi.org/10.6084/m9.figshare.22734758.

**FIGURE 1:**
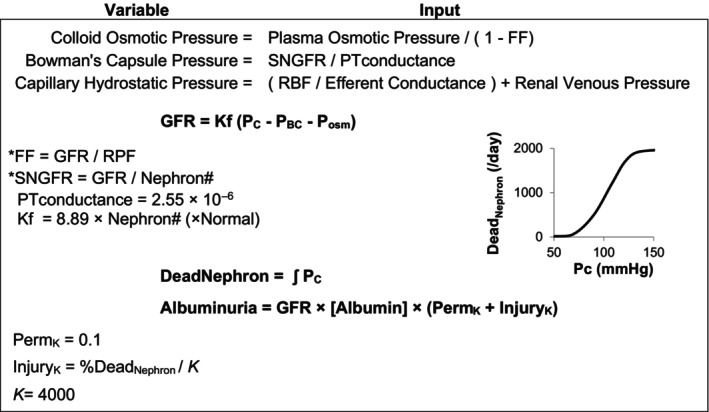
Equations and model parameters for calculating glomerular filtration rate and nephron injury. FF, filtration fraction; SNGFR, single nephron glomerular filtration rate; PTconductance, conductance of the proximal tubule; RBF, renal blood flow; Kf, filtration coefficient; P_C_, capillary hydrostatic pressure; P_BC_, Bowman's Capsule hydrostatic pressure; P_osm_, capillary colloid osmostic pressure; RPF, renal plasma flow; and Perm_K_, permeability constant. Excretion of albumin is dependent on glomerular filtration, plasma albumin concentration, nephron injury, and baseline permeability which is set to 0 in normal conditions. * indicates implicit equation.

The model integrates multiple physiological systems, including those most relevant to this study, that is, renal, endocrine, body fluid compartments, and cardiovascular systems and is based on the interrelationships of fluid volumes and pressures through changes in pressures, flows, and the renal excretion of sodium and water (Guyton, 1980, 1990; Guyton et al., 1972). In brief, the kidneys in HumMod are comprised of vascular and tubular components. Glomerular filtration is influenced by physical factors such as hydrostatic and osmotic pressures (Figure 1). For the current study, we modeled the progression of hypertensive nephrosclerosis, the second leading cause of ESRD and mechanistically attributed to glomerular HTN (Costantino et al., 2021). The rate of nephron loss and rate of albuminuria were largely determined by glomerular capillary pressure (Figure 1) and by increasing baseline glomerular permeability to albumin to reach levels similar to the lower range (non‐diabetic end) of the EMPA‐KIDNEY and DAPA‐CKD trials (Heerspink et al., 2020; The et al., 2023). Nephron death was set to occur at a glomerular pressure above 70 mmHg based on the function shown in Figure 1. This function and related parameters were matched to the decline in renal function observed in the placebo arm of the EMPA‐KIDNEY and DAPA‐CKD trials (Figure S1) and corresponding to the 10%–20% increase in glomerular pressure needed to induce proteinuria and glomerular injury in animal CKD models (Fan et al., 2021; Tapia et al., 2012).

**FIGURE 2:**
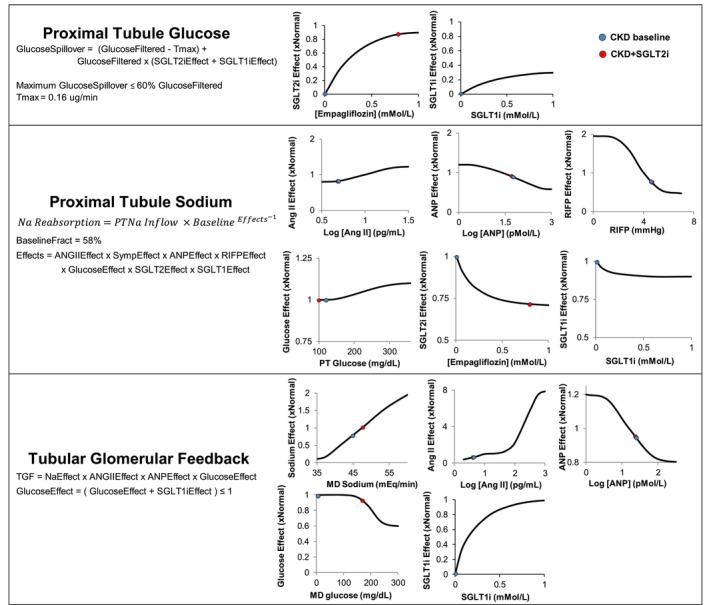
Model relationships for the impact of glucose, SGLT1, and SGLT2 inhibitor within the kidney. SGLT1 and SGLT2, sodium‐glucose cotransporter 1 and 2. Redmarker indicates values corresponding to the CKD+SGLT2i simulation after 25 mg empagliflozin during maximum concentration. Primary effects (based on empagliflozin concentration) were on proximal tubular sodium and glucose reabsorption. Tmax indicates transport maximum for glucose reabsorption in individualproximal tubules; SGLT, sodium glucose cotransporter; SGLT1i, sodium glucose cotransporter‐1 inhibitor; PTNa, proximal tubular sodium; baseline fract, baselinefractional reabsorption; ANG II, angiotensin II; Symp, sympathetic; ANP, atrial natriuretic peptide; RIFP, renal interstitial fluid pressure; and MD, macula densa. The effect of sympathetic nerve activity on proximal tubular sodium reabsorption is not shown. MD glucose is normally 0 mg/dL.

Complete proximal tubular reabsorption of glucose was assumed unless filtered glucose rose above 0.16 μg/min (corresponding to 375 mg/min) or if there was SGLT2 inhibition (with or without SGLT1 inhibition) (Figure 2). The maximum glucose spillover per nephron was assumed to be 60% based on studies in healthy humans with high‐dose empagliflozin (Seman et al., 2013). Proximal tubular sodium reabsorption was determined by a baseline fractional reabsorption (58%) that could be influenced by factors including plasma angiotensin II, atrial natriuretic peptide (ANP), renal interstitial fluid pressure, tubular glucose concentration, and SGLT2/1 inhibition (Figure 2). The relationship between glucose and sodium reabsorption is based on rodent and computer simulation data showing 20 mM glucose increases proximal tubular sodium reabsorption by 10% (Layton et al., 2015). SGLT2 inhibition of sodium reabsorption (up to ~30%) is based on chronic micropuncture studies in diabetic rats (Thomson et al., 2012). The ability of SGLT1 inhibition to decrease proximal tubular sodium reabsorption (up to 10% during SGLT2 blockade) was based on the compensation that occurs chronically in SGLT2 knock out mice (Vallon et al., 2011).

Renal hemodynamics and tubular functions are influenced by physical factors, the sympathetic nervous system, circulating hormones, and TGF. TGF is primarily determined by the delivery of filtered sodium to the macula densa, but also by angiotensin II and ANP (both minor effects) (Figure 2). Additionally, filtered glucose both indirectly and directly affected TGF through its impact on sodium delivery to the macula densa and through the macula densa SGLT1 pathway, respectively. The SGLT1 relationship was based on renal micropuncture experiments in mice demonstrating hyperglycemia in the tubular lumen (17 mM glucose) enhanced vasodilation in the afferent arteriole (40%) with SGLT1 inhibition blocking this effect (Zhang et al., 2019). In the model, SGLT1 inhibition blocks this effect as shown in Figure 2 and demonstrated in the CKD + SGLT2/1 simulation.

Plasma levels of hormones are calculated from rates of secretion/production, clearance rates, and volume of distribution. Key determinants of renin secretion are β‐adrenergic stimulation of juxtaglomerular cells and sodium chloride delivery to the macula densa, the latter influenced by both RSNA and ANP (Figure S2). The primary determinants of aldosterone secretion are blood concentrations of angiotensin II (ANG II) and potassium. Organs and tissues that make up the circulation in HumMod include the kidneys, heart, skeletal muscle, gastrointestinal tract, liver, bone, brain, fat, skin, and lungs. The flow through these tissues is affected by sympathetic activity, plasma ANG II, and local tissue factors such as oxygen, which together regulate vascular tone and organ blood flow.

### 2.2 Empagliflozin model

**FIGURE 3:**
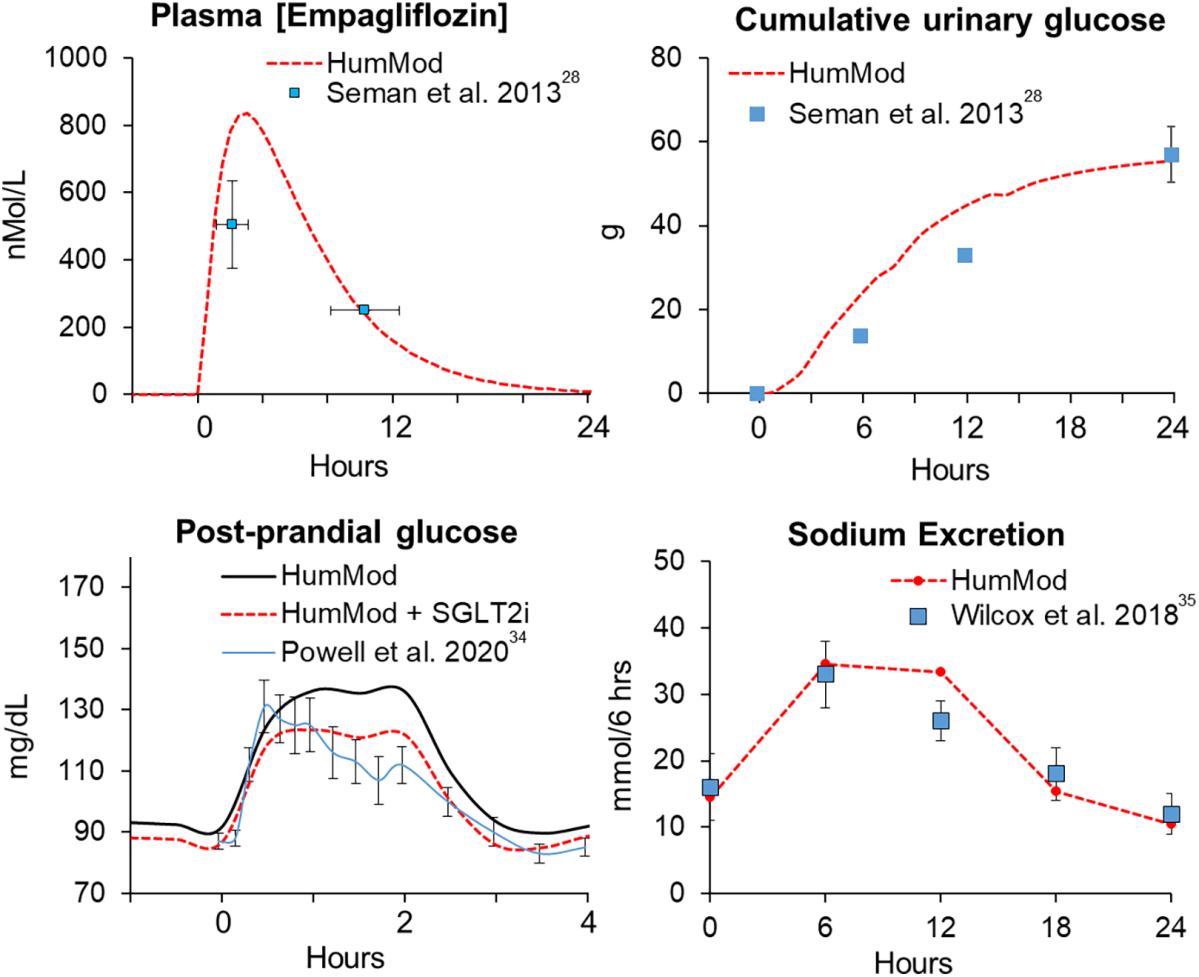
Responses to single dose of SGLT2i in normal healthy adults and in HumMod with normal renal function. Clinical data shown in mean ± STD. SGLT2, sodium‐glucose cotransporter 2.

Bioavailability of empagliflozin in the model was assumed 78% (Ndefo et al., 2015). Maximum concentration (505 ± 130 nmol/L) and plasma half‐life (10 ± 2 h) after 25 mg empagliflozin were used to compare single‐dose studies in healthy subjects (Seman et al., 2013) (Figure 3). Also, maximum empagliflozin concentration in the model (830 nmol/L) was within range with a study examining normal fasted subjects (700–1400 nmol/L) (Glund et al., 2017). Cumulative urinary glucose responses from this study were also used to validate the SGLT2 inhibitor model (Figure 3). Post‐prandial plasma glucose responses from healthy subjects treated with canagliflozin were used to confirm similar plasma glucose levels in the model that are seen in normoglycemic subjects treated with SGLT2 inhibition (Powell et al., 2020). Finally, urinary sodium excretion in response to a single dose of a SGLT2 inhibitor were compared to responses in healthy normoglycemic subjects (Wilcox et al., 2018) (Figure 3).

### 2.3 Protocols

To mimic experimental conditions of CKD, right and left kidney functional nephron number was reduced to 25% of baseline (300,000 nephrons per kidney). Glomerular permeability to albumin was also increased to give significant baseline albuminuria (500 mg/day) (Table 1). These conditions were simulated for another 6 months to reach steady‐state while dietary food intake was held constant and water intake was ad libitum. Next, a 3 meal/day schedule was simulated (2000 kcal, 60% carbohydrate, 25% fat, and 15% protein) for 1.5 months to allow model variables to reach a steady‐state. All simulations had sodium and potassium intake fixed at normal levels (180 and 70 mmol/day, respectively).

**TABLE 1:** Cardiovascular, renal, and fluid responses to chronic kidney disease and SGLT2 inhibition.

	Baseline		Acute (2 h)		Chronic (1 year)		
	Normal	CKD	+SGLT2i	+SGLT1/2i	+SGLT2i	+SGLT1/2i	No treatment
SBP (mmHg)	127	152	152	152	149	150	154
MAP (mmHg)	94	110	110	110	107	108	110
DBP (mmHg)	75	84	85	85	82	83	84
HR (bpm)	62	62	62	62	62	62	62
Nephron # (million)	2.39	0.579	0.579	0.579	0.526	0.535	0.511
[SGLT2i] (nmol/L)	0	0	827	827	268	268	0
GFR (mL/min)	115	55	52	50	51	50	51
Albuminuria (mg/day)	0	499	482	462	519	503	547
Chronic GFR decline (mL/min/yr)	1.0	–	–	–	2.8	2.1	3.6
RBF (mL/min)	1106	491	474	461	460	458	455
Aff Art R (mmHg/mL/min)	0.034	0.062	0.069	0.075	0.062	0.066	0.064
Glomerular pressure (mmHg)	56	78	76	75	78	77	80
SNGFR (nl/min)	48	95	91	87	97	94	100
Na reabsorption (mmol/min)	16	7.5	7.1	6.8	6.9	6.9	7.0
Na excretion (mmol/day)	100	180	207	206	180	180	180
FENa (%)	0.41	1.64	1.98	2.06	1.77	1.78	1.75
Glucose excretion (g/day)	0	2	23	22	22	22	3
Glucose excretion (mmol/L)	0	0.02	0.9	0.8	0.42	0.40	0.04
FEGlucose (%)	0	1.19	60	60	26	28	2
Fasting glucose (mg/dL)	92	94	89	89	91	91	95
Post‐prandial glucose (mg/dL)	136	142	136	128	135	129	148
24‐hr glucose (mg/dL)	109	114	–	–	112	112	116
PV (L)	3.2	3.6	3.5	3.5	3.6	3.6	3.7
ECFV (L)	15.0	15.6	15.4	15.4	15.5	15.5	15.7
RAP (mmHg)	1.0	1.8	1.4	1.4	1.7	1.8	2.0
ANP (pmol/L)	26	47	39	38	43	44	53
NE (pg/mL)	238	237	238	238	237	237	237
ANG II (pg/mL)	11	5	5	5	5	5	5

After recording 1.5 months of baseline values with 3 high carbohydrate meals per day, responses to 1 year of SGLT2 inhibition were determined for each of the following simulations:

1. *CKD*: Low renal function (25% nephrons remaining)
2. *CKD + SGLT2i*: CKD model with 25 mg empagliflozin/day with only inhibitory capabilities on SGLT2
3. *CKD + SGLT1/2i*: CKD model with 25 mg empagliflozin/day with inhibitory capabilities on both SGLT1 and SGLT2 activity.

Additional simulations determining the effect of changing carbohydrate or salt intake on chronic CKD responses are provided in the supplement (Figures S3 and S4, respectively).

## 3 RESULTS

Baseline conditions and acute and chronic responses to SGLT2 inhibition are summarized in Table 1. Baseline CKD was associated with HTN (152/84 mmHg), glomerular HTN (78 mmHg), albuminuria (405 mg/g creatinine), normal glucose levels, but slight glycosuria (0.02 mmol/L) (Table 1). After 1 year, CKD without treatment was associated with a reduction in nephrons (−11%), lower GFR (−3.6 mL/min), sustained glomerular HTN (80 mmHg), and albuminuria (445 mg/g) compared to baseline.

**FIGURE 4:**
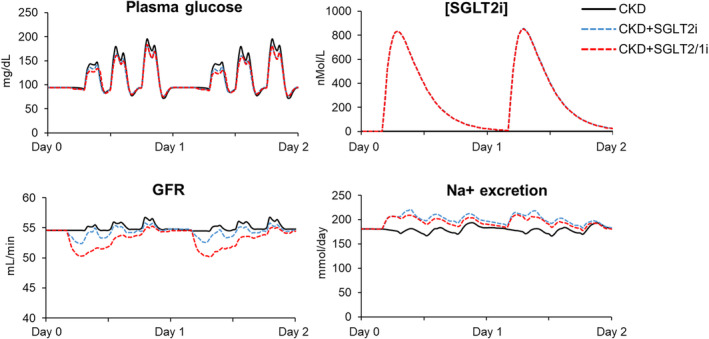
Acute responses to SGLT2i during high carbohydrate diet and CKD. CKD control simulation is indicated with a solid black line. SGLT2 inhibition is shown with a dashed blue line and the combination SGLT2 and SGLT1 inhibition simulation is indicated by a dashed red line. CKD, chronic kidney disease; SGLT1 and SGLT2, sodium‐glucose cotransporter 1 and 2.

**FIGURE 5:**
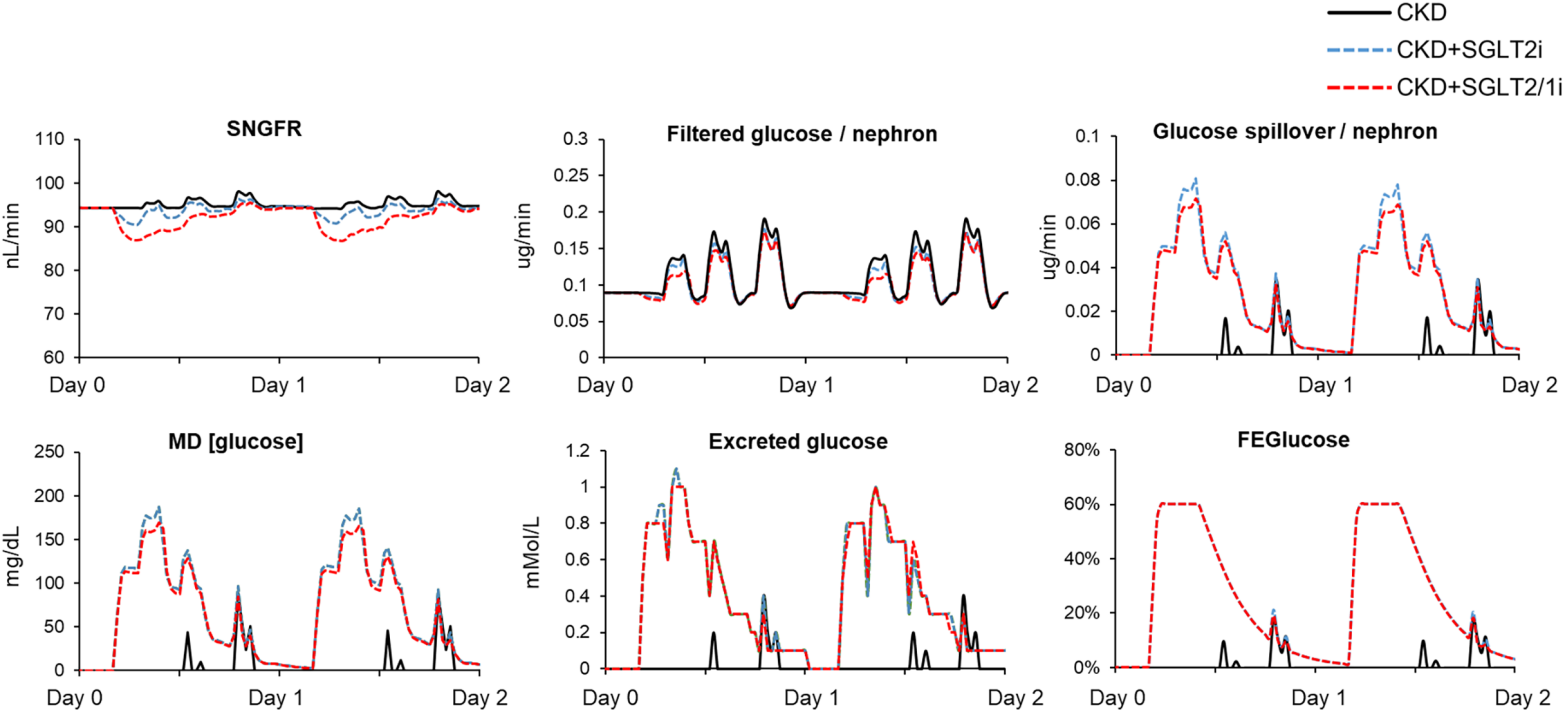
Glucose handling in individual nephrons after first 2 days of SGLT2 inhibition in the conditions shown in Figure 4. SGLT2, sodium‐glucose cotransporter 2.

After 25 mg empagliflozin, there were no changes in systemic BP but declines in GFR, RBF, glomerular pressure, and Na^+^ reabsorption at 2 h (Table 1, Figure 4). There were also increases in glucose excretion (23 g/day) but little change in plasma glucose levels (Figures 4 and 5). Simulating additional SGLT1 inhibitory effects were associated with lower post‐prandial glucose (128 vs. 136) and greater reductions in SNGFR (−8% vs. −4%) as compared to SGLT2 inhibition alone (acutely). Due to these changes (slightly lower plasma glucose and greater reduction in SNGFR), the SGLT1 simulation was associated with similar glycosuria as compared to the SGLT2 simulation (Figure 5).

**FIGURE 6:**
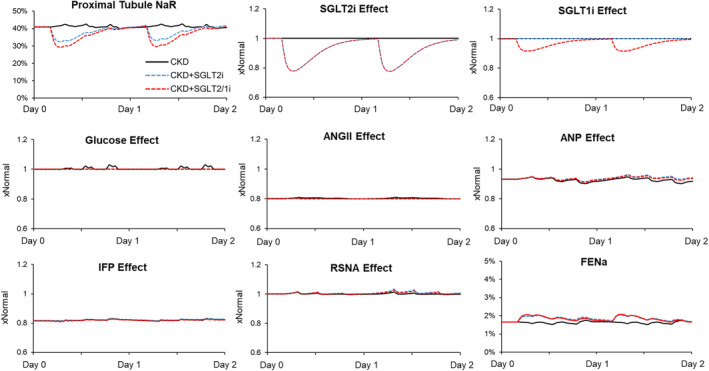
Determinants of proximal tubular sodium reabsorption in CKD during first days of SGLT2 inhibition in the conditions shown in Figure 4. CKD, chronic kidney disease; SGLT2, sodium‐glucose cotransporter 2.

Determinants of proximal tubular sodium reabsorption with and without SGLT2 inhibition are shown in Figure 6. At baseline, CKD simulation was associated with lower proximal tubular fractional Na^+^ reabsorption (41%) as compared to normal (58%) resulting from suppressed angiotensin II, increased atrial natriuretic peptide, and high renal interstitial fluid pressure (Figure 6). SGLT2 inhibition acutely decreased fractional proximal tubular sodium reabsorption (−8%). When simulating additional SGLT1 inhibition, this was further reduced (−12%) although this resulted in relatively similar increases in the fractional excretion of Na^+^ (Figure 6).

**FIGURE 7:**
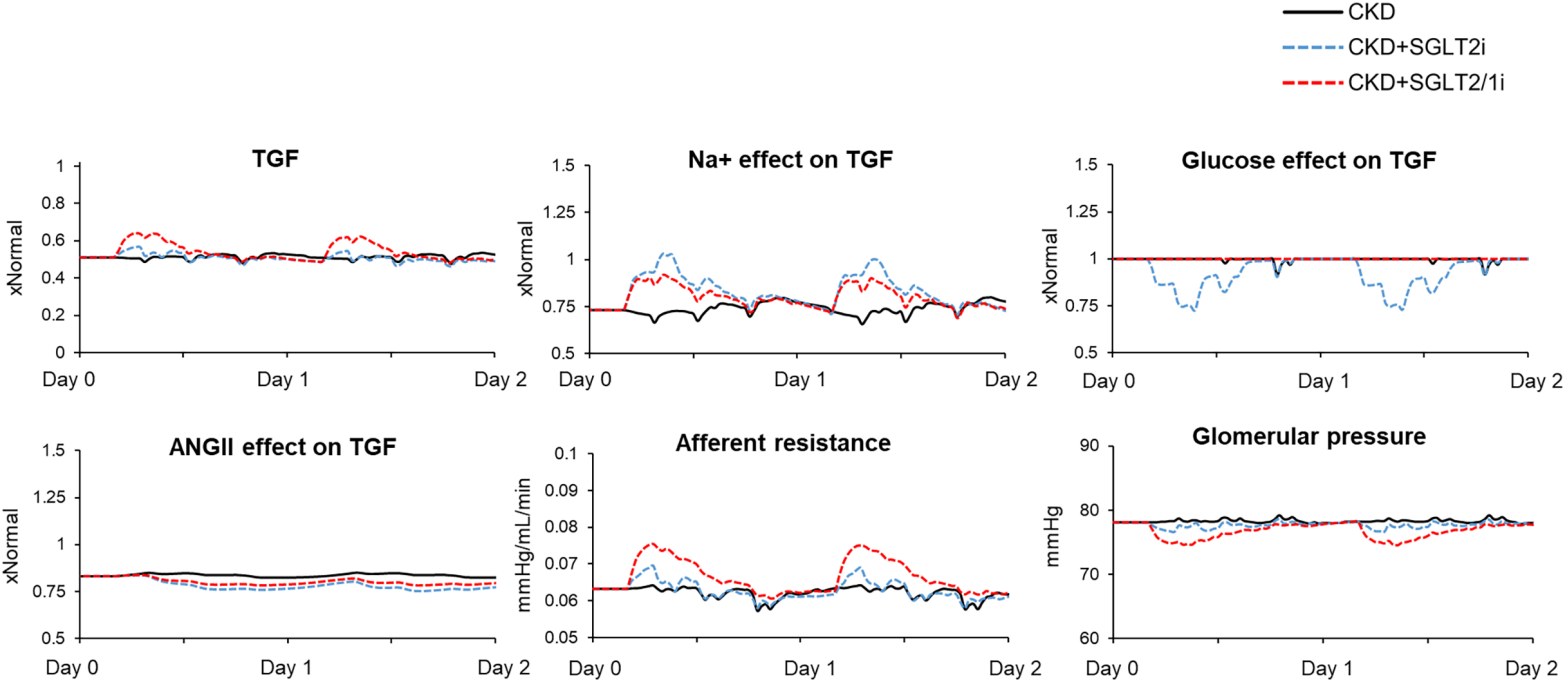
Determinants of tubuloglomerular feedback and acute changes in glomerular hemodynamics after first days of SGLT2 inhibition in the conditions shown in Figure 4. SGLT2, sodium‐glucose cotransporter 2.

Figure 7 shows the determinants of TGF. As compared to normal, baseline CKD was associated with lower activation of TGF due to effects from Na^+^ at the macula densa and lower angiotensin II (Figure 7). In response to SGLT2 inhibition, there were acute increases in sodium delivery to the macula densa, resulting in increased TGF (Figure 7). In parallel, the increased glucose delivery to the macula densa contributed a TGF‐lowering effect. Despite this inhibitory effect from glucose spillover during SGLT2 inhibition, afferent resistance still acutely increased, leading to a reduced glomerular pressure (−2%, Figure 7). Simulating SGLT1 inhibition with SGLT2 inhibition resulted in a slight decrease in sodium delivery to the macula densa (associated with greater reductions in single nephron GFR), but there was complete blockade of the glucose effect on TGF (Figure 7). Overall, this resulted in greater afferent arteriolar vasoconstriction and a further decrease in glomerular pressure (−4%).

**FIGURE 8:**
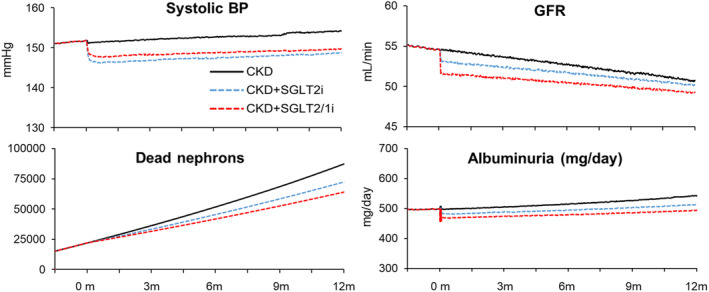
Blood pressure and renal responses to chronic (12 months) SGLT2 inhibition in simulations described in Figure 4. SGLT2, sodium‐glucose cotransporter 2.

Chronic effects of SGLT2 inhibition on BP and renal function are shown in Figure 8. Over 12 months, untreated CKD was associated with 88 k dead nephrons and albuminuria (547 mg/day). As compared to CKD without treatment, 12 months of SGLT2 inhibition was associated with a 5 mmHg lower systolic BP (Figure 8), relatively similar GFR, 18% reduction in dead nephrons, and 5% reduction in albuminuria (Figure 8). SGLT2/1 inhibition was associated with a 4 mmHg lower systolic BP at 1 year and reductions in dead nephron count and albuminuria (−28% and −8%, respectively), as compared to untreated CKD (Figure 8).

## 4 DISCUSSION

While not specifically designed for modeling CKD, HumMod is able to accurately simulate many pathophysiological features of CKD as well as replicate the acute and chronic physiological responses to SGLT2 inhibition in non‐diabetic settings (Figure 3, Figure S1). In the present study, our simulations integrated physiological responses to the daily undulation of salt, glucose, and drug concentrations. Also, the model's predictions demonstrate that SGLT2 inhibition decreases the chronic rate of decline in GFR and reduce onset of albuminuria in normoglycemic CKD through improvements in glomerular HTN via TGF. This occurred despite the increased delivery of glucose to the macula densa which could have potentially worsened hyperfiltration. After adding SGLT1 inhibitory effects, these renoprotective benefits were enhanced, suggesting SGLT1/2 combination blockade may be especially beneficial in non‐diabetic CKD.

Clinically, the renoprotection during SGLT2 inhibition seems to be independent of etiology or severity of CKD, age, gender, or presence of heart failure (Heerspink et al., 2020; The et al., 2023). There are many postulated mechanisms underlying the renoprotective effects of SGLT2 inhibitors including improvements in renal oxygenation and inflammation (Heerspink et al., 2019), reducing HIF‐1a (Packer, 2021), and blocking hyperfiltration via TGF (Thomson & Vallon, 2021). The extent of this renoprotection that can be explained by glomerular hyperfiltration/proteinuria reduction through TGF feedback or by BP control has not been quantified. Recently, in a normoglycemic 5/6th nephrectomy rat model, there was increased BP and glomerulosclerosis associated with decreased TGF (as assessed by urinary adenosine) (Chen et al., 2023). Interestingly, SLGT2 inhibition significantly increased TGF and abolished the increases in renal fibrosis and proteinuria (Chen et al., 2023). While TGF cannot be assessed directly in humans, this evidence along with the current simulations suggest a critical role for SGLT2i in ameliorating the progression of non‐diabetic CKD via activation of TGF.

Our current study suggests when paired with a relatively normal salt diet, SGLT2 inhibitors do not elicit a chronic RAS response, even in the absence of RAS inhibitors, similar to findings in healthy subjects (Isshiki et al., 2020) and experimental studies in a rat model of CKD (Li et al., 2018). Some studies in T2D note small initial increases in RAS after acute SGLT2 inhibition (Puglisi et al., 2021), but the chronic RAS responses to SGLT2 inhibition have not been thoroughly investigated. As alluded to earlier, RAS blockade exists in virtually all SGLT2 inhibitor trials in CKD patients, suggesting separate mechanisms and an additive benefit with these two critical treatments of CKD. Indeed, RAS inhibitors have been shown to prevent CKD progression, even compared to other antihypertensive therapies with similar BP lowering effects, most likely through lowering of glomerular pressure via efferent arteriolar vasodilation (Hou et al., 2006; Wright Jr et al., 2002). Similarly, chronic angiotensin receptor blockade in the current CKD model resulted in the reduction of efferent resistance, glomerular pressure, albuminuria, and chronic GFR decline; and importantly, the addition of SGLT2 inhibition further improved these parameters (Figure S5).

Urinary albuminuria is a commonly used (and easily accessible) short‐term marker for glomerular pressure and to gauge the beneficial responses to renoprotective treatments such as RAS inhibitors. The current model predictions suggest SGLT2 reduces albuminuria 5%–10% acutely and is sustained for 12 months (Figure 8). Clinically, SGLT2 inhibitors lower albuminuria by 15% (6% to 23%, 95% CI) in non‐diabetic CKD within the first 2 weeks and persists up to 3 years of follow‐up (Jongs et al., 2021). Results from the EMPA‐Kidney trial demonstrate that CKD patients with higher albuminuria may benefit more from SGLT2 inhibition (The et al., 2023). Some of this reduction is most likely hemodynamically related. For example, of the total 25% reduction in urinary albumin in T2D patients treated with a SGLT2 inhibitor, approximately 10% was attributable to the reduction in BP (Cherney et al., 2017). However, 3 years of SGLT2 inhibitor treatment was associated with 29% less albuminuria than the placebo group event after individuals were taken off the treatment, suggesting permanent improvements in renal function (Cherney et al., 2017). Similarly, it was only recently demonstrated that chronic SGLT2 inhibition can provide structural changes in the nephron in normoglycemic animals, as shown by decreases in glomerular and tubulointerstitial fibrosis (Chen et al., 2023). This evidence paired with our model's prediction of 18 and 28% reductions in dead nephrons in the SGLT2 and SGLT2/1 simulations, respectively, suggest key mechanisms of SGLT2 renoprotection in CKD is through both acute reduction in glomerular pressures and chronic structural protection of functional nephrons.

The only large randomized trial investigating the impact of SGLT2/1 inhibition (sotagliflozin) in CKD was performed recently in diabetic individuals with or without albuminuria (Bhatt et al., 2021). This study demonstrated SGLT2/1 inhibition reduced risk of the composite of the total number of deaths from cardiovascular disease, hospitalization for heart failure, and urgent visits for heart failure. While the study did not find statistical significance in the renal composite outcome, the estimated hazard ratio was approximately 0.7, which is consistent with, but not stronger than, findings from SGLT2 inhibitor trials. While it is difficult to extrapolate these results and apply them to the non‐diabetic CKD setting, our current study suggest that SGLT2/1 inhibition may provide additional renoprotection, especially when diabetic ketoacidosis is not a concern (Bhatt et al., 2021).

The current CKD model was associated with low levels of post‐prandial glycosuria at baseline (0.02 mmol/L). Mild glycosuria has been noted in CKD patients. Causes and consequences of glycosuria in non‐diabetic CKD is not well studied. Increased fractional excretion of glucose occurs with more advanced stages of CKD due to tubular dysfunction and is associated with greater proteinuria (Hung et al., 2016). Using the current model, we demonstrate that a low carbohydrate diet (40% of total caloric intake) was associated with very modest improvements in post‐prandial glucose levels but a 5% reduction in dead nephrons after a year (Figure S3). The possible benefits of a low carbohydrate diet in patients with CKD can be difficult to demonstrate in clinical studies due to its promotion of ketoacidosis in patients with diabetes and due to the dietary restrictions of high protein‐based diets in patients with low renal function (Mitchell et al., 2020). Recommended carbohydrate intake in the diet is 45%–65%, and the average carbohydrate intake is around 50% for US adults (Shan et al., 2019). Interestingly, in CKD patients, low carbohydrate diets such as a Mediterranean or plant‐based diets are associated with lower mortality and decreased risk of ESRD (Gutierrez et al., 2014; Huang et al., 2013).

There are limitations to the current study. First, lowering glomerular pressure can improve factors such as inflammation, endothelial dysfunction, and oxidative stress, but these factors were not modeled in the current study. Other potential determinants of renal function that currently are also not incorporated in the model (e.g., nitric oxide, reactive oxygen species, or cytokines) may need to be included in future models to better understand the cardiovascular responses to SGLT2 inhibition. Third, SGLT2 inhibition may exert additional renal benefit through weight reduction in the obese population, but this weight loss is usually modest (1 kg over 2 years in CKD patients) (The et al., 2023). Fourth, HumMod does not account for the glomerular hypertrophy or compensation that may occur to individual proximal tubules during chronic hyperfiltration. Finally, future efforts should investigate the benefits of SGLT2 inhibitor therapy in other understudied pathophysiological states such as in advanced CKD and CKD with concomitant heart failure. Indeed, the reduced risk of heart failure that has been associated with SGLT2 inhibition may also positively impact renal outcomes.

## FUNDING INFORMATION

This work was supported by grants from the National Institute on Minority Health and Health Disparities (R00 MD014738) and National Institute of General Medical Sciences (U54 GM115428) to JSC and the National Institute of General Medical Sciences (P20 GM104357) to the UMMC Department of Physiology.

## CONFLICT OF INTEREST STATEMENT

The authors declare that there are no competing interests.

## DISCLOSURES

None.

## Supporting information


Appendix S1:
