# Supplementary material for: Modeling the renoprotective mechanisms of SGLT2 inhibition in hypertensive chronic kidney disease
Source: Physiol Rep. 2023 Nov 13;11(21):e15836. doi: 10.14814/phy2.15836 (PMC10643202; doi:10.14814/phy2.15836)

Supplementary Figure S1. Comparison GFR changes during chronic kidney disease with and without SGLT2i treatment in the HumMod model and the DAPA-CKD Trial

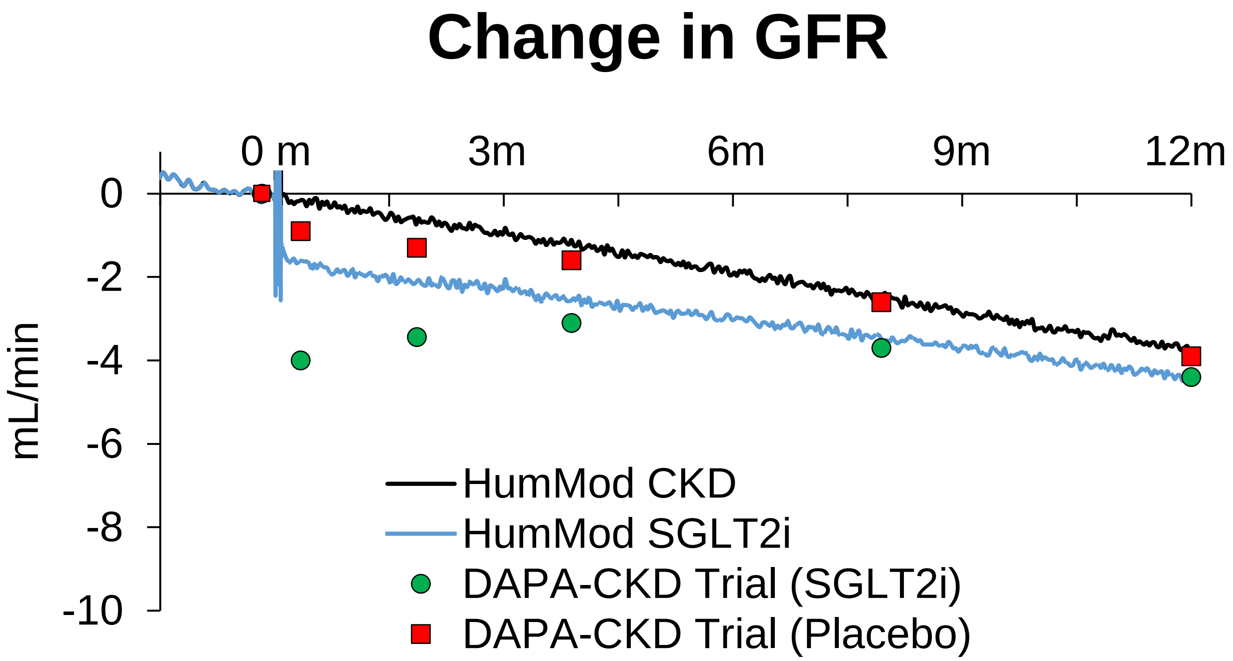

Supplementary Figure S2. Control of renin secretion and angiotensin II in response to SGLT2 inhibition. Renin secretion is a function of macula densa signal, sympathetic nervous activity, and renin mass. When simulating chronic kidney disease (25% remaining nephrons), renin mass is decreased 75%.

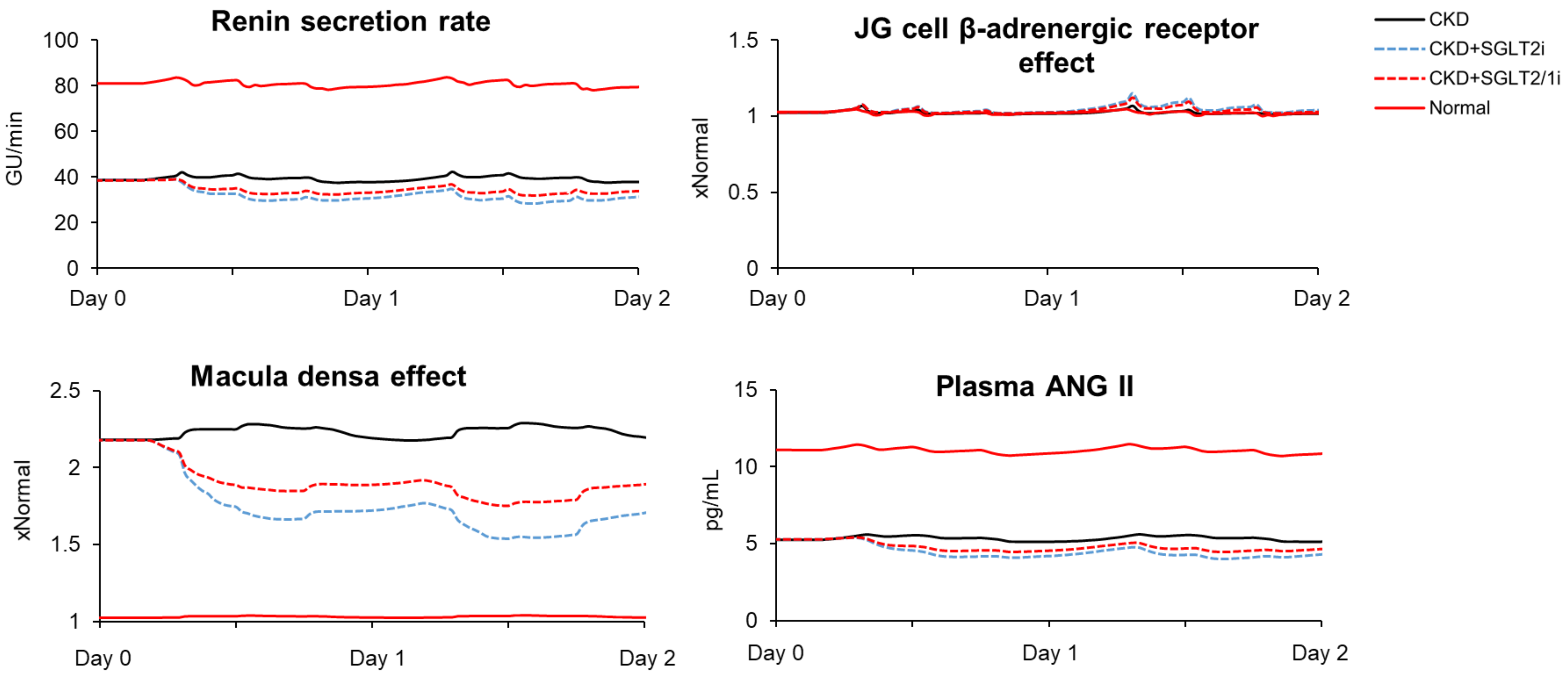

Supplementary Figure S3. Effects of chronic (1 year) carbohydrate intake in CKD model. Low, normal, and high carbohydrate intakes corresponded to 40%, 50%, and 60% of total diet. After 1 year, the 40% diet was associated with a 1% and 3% reduction in albuminuria and GFR decline, respectively.

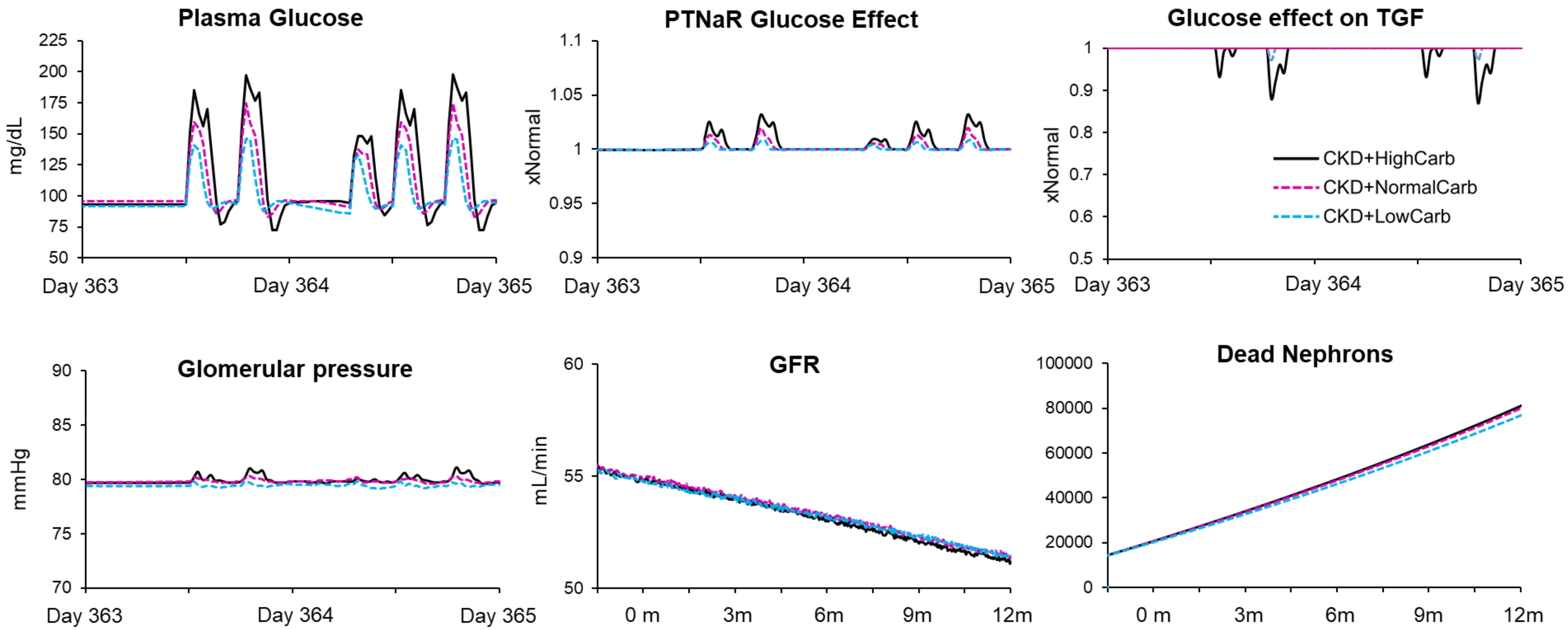

Supplementary Figure S4. Acute and chronic responses to SGLT2 inhibition with and without the addition of 70 mmol of Na+ (250 mmol/day total). High Na+ diet increased baseline glomerular filtration rate (GFR) but also glomerular pressure, blood pressure, nephron damage, and worsened the chronic decline in GFR.

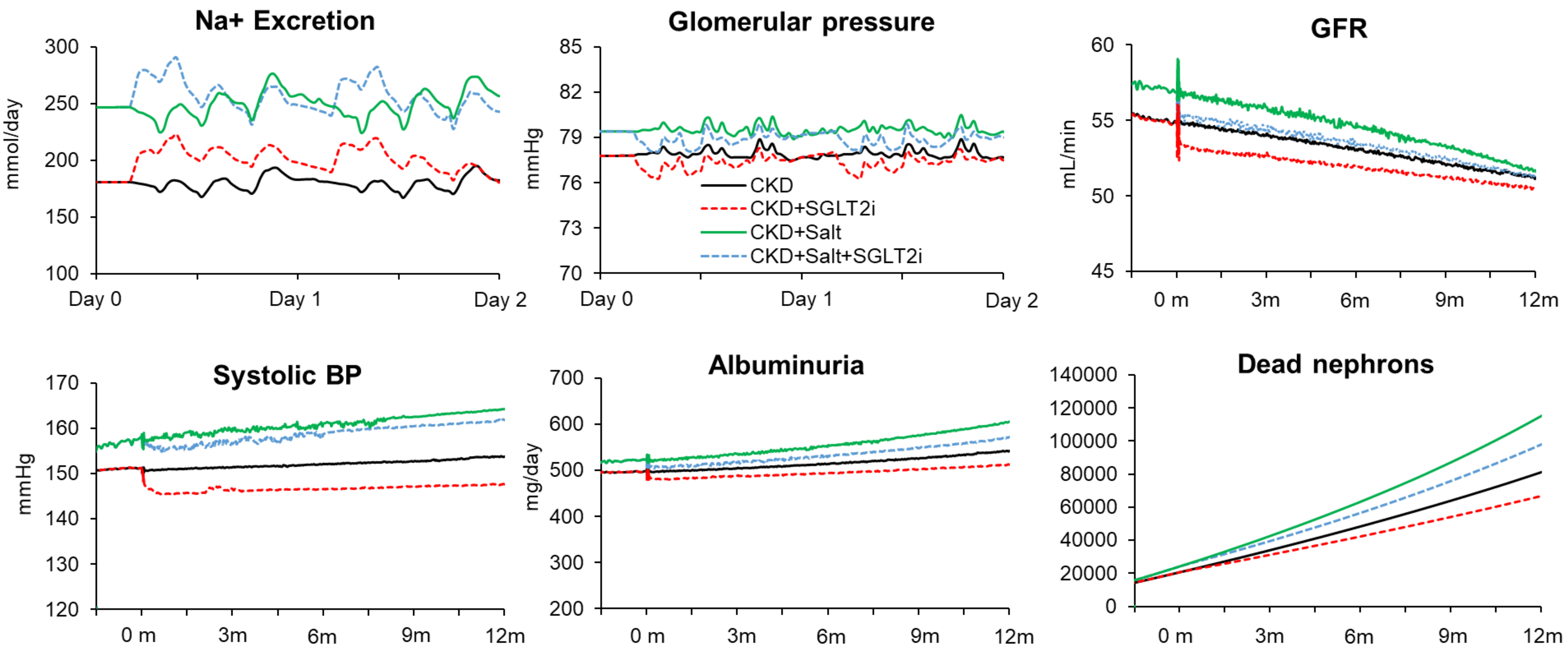

Supplementary Figure S5. CKD with and without existing angiotensin receptor blocker therapy (50 mg/day losartan) and responses to SGLT2 inhibition (25 mg/day empagliflozin). The chronic slope for the change in glomerular filtration rate (GFR) was -3.6, -2.8, -2.8, and -2.3 mL/min/yr for the CKD, CKD+ARB, CKD+SGLT2i, and CKD+SGLT2i+ARB simulations, respectively.

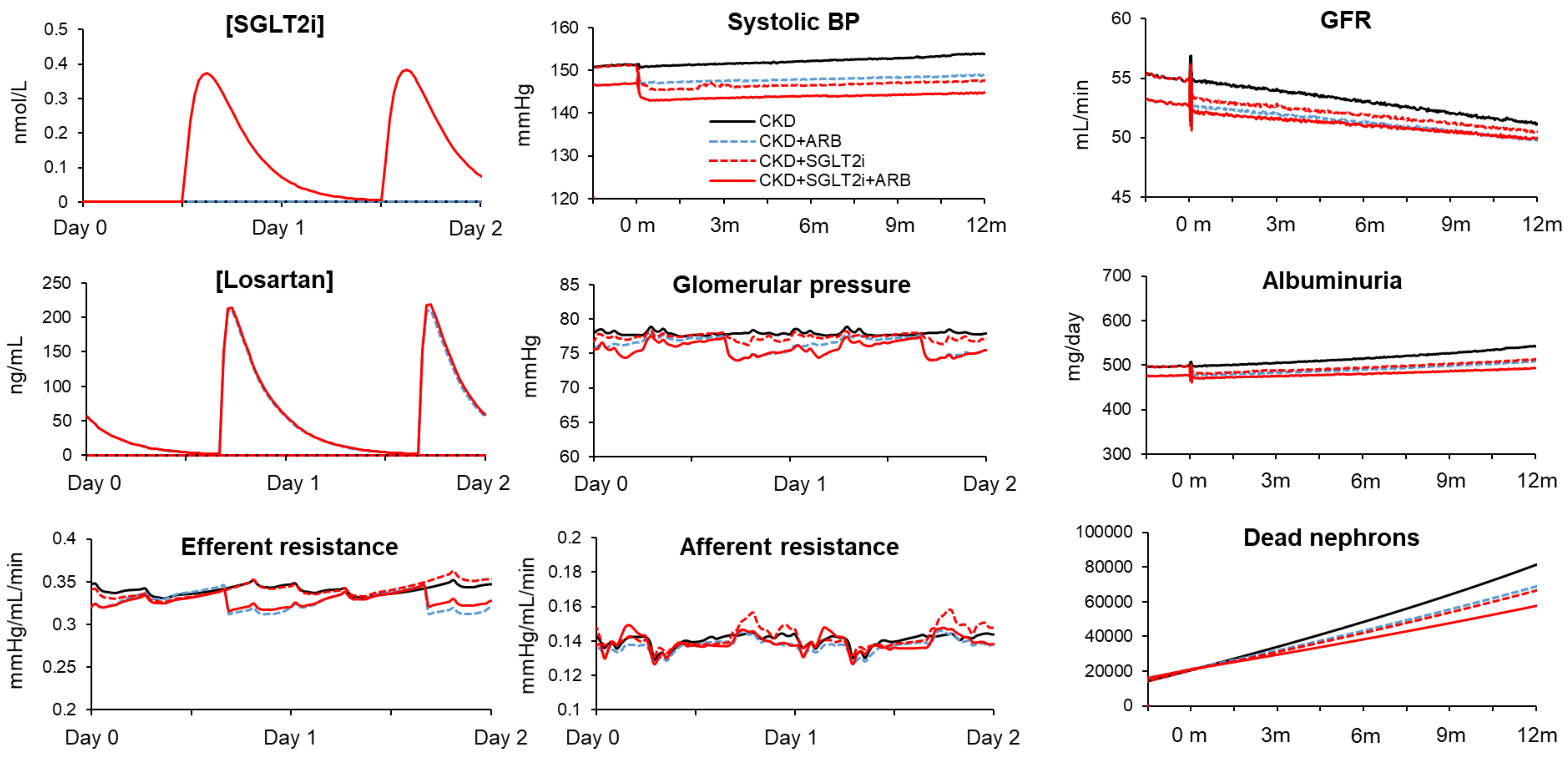

Supplement: Supplementary file 1 — Appendix S1: [file PHY2-11-e15836-s001.pdf]
